# Can Physical Examination Skills Survive the Era of Modern Diagnostic Tests?

**DOI:** 10.15694/mep.2017.000003

**Published:** 2017-01-09

**Authors:** Mohammed Elhassan

**Affiliations:** 1 UCSF/Fresno Center for Medical Education and Research

**Keywords:** Physical Examination Skills, Likelihood ratio

## Abstract

This article was migrated. The article was marked as recommended.

There is a perception by the internal medicine community that physical examination skills have declined amongst both learners and teachers of medicine. A sense of lack of time and the development of more sensitive and specific diagnostic tests are amongst some of the reasons for this phenomenon. Internists need to be familiar with the clinical usefulness of physical examination maneuvers learned during medical school and residency training so that they know, at the bedside, what to focus on during the patient encounter. Here I would like to highlight some of these easy to perform physical examination signs that some data argues for and support their clinical utility in daily practice in the era of modern diagnostic technology. These are: the third heart sound; maximal tracheal height and Hoover’s sign; the “HINTS” examination for patients with vertigo; and simple bedside questions to screen for delirium.

## Introduction

There is a perception by the internal medicine community that physical examination skills, for many different reasons, have declined amongst both learners and teachers of medicine. Moreover, it is not an infrequent scenario for internists seeing patients in in-patient or out-patient setting to feel rushed at the bedside as they are trying to deliver high-value care for their patients. Many of the physical examination signs can be tested, just like any other diagnostic test, for their validity and power to rule in or rule out a disease. Internists need to be familiar with the clinical usefulness of physical examination maneuvers learned during medical school and residency training so that they know, at the bedside, what to focus on during the patient encounter. Here I would like to highlight some examples of bedside maneuvers which data support their utility at the bedside. They are also relatively simple to perform and thus can help when time constrain is an issue. They are: the third heart sound; maximal tracheal height and Hoover’s sign; the “HINTS” examination for patients with vertigo; and simple bedside questions as part of the mental status examination to screen for delirium.

## Third heart sound

A common reason for hospital admissions or a call to the in-house physician is evaluation of patient with acute onset or worsening of short of breath (SOB), where acute heart failure is usually one of the main considerations in the differential diagnosis. The examining physician might order a serum B-natriuretic peptide (BNP) and/or chest radiograph besides other tests to try to determine the etiology of SOB. Most physicians in these circumstances will also try to listen carefully to the presence or absence of a third heart sound (S3) during cardiac auscultation. Although not quite sensitive, S3 has shown good specificity and positive predictive value for detecting low ejection fraction and increased end diastolic volume. In one study ( Marcus, 2004), an S3 has a positive likelihood ratio (LR+) of 10 for high BNP (i.e. increases probably of a high BNP test by about 45%). Although probably nowadays not too many examiners will try to elicit S3 by turning the patient to the left decubitus position and listen again with the bell, rather than the diaphragm, of the stethoscope, it seems reasonable to try to make that extra effort to elicit this sign. In one study ( Bethell, 1973), turning the patient to the lateral decubitus position increased the audibility of S3 from 8 to 17 patients and of any gallop from 28% to 90% of patients. Thus, it can be a useful adjunct to bedside evaluation before tests like serum BNP is back or even without ordering it. This will also increase your confidence to initiate therapy, in this case diuretics, early. Also, eliciting S3 during the evaluation of SOB after non-cardiac surgery was found to correlate well with post-operative pulmonary edema with a LR+ of 14.6 and also with post-operative myocardial infarction with a LR + of 8 ( Goldman, 1977).

## Maximal tracheal height and Hoover’s sign

Presence of wheezes significantly increases the probability of airway obstruction as most physicians expect, but it is not uncommon for acute heart failure to be associated with wheezes also, sometimes called “cardiac asthma”. Two relatively helpful signs are observation of short tracheal height (4 cm or less) and inspiratory inwards movement of the lower intercostal spaces bilaterally (i.e. “Hoover’s sign”). Observing that the laryngeal height (distance between the top of the thyroid cartilage and the suprasternal notch) is short, specifically 4 cm or less, has a LR+ of 3.6 to support the diagnosis of obstructive airway disease ( Straus, 2000). In the other hand, in one study ( Garcia-Pachon, 2002), presence of Hoover’s sign, which is not uncommon observable chest wall abnormality, increases the probability of airway obstruction by about 25-30% with a LR+ of 4.2. Both of these physical findings are thought to be due to increased diaphragmatic descent during inspiration when there is limitation of air movement due to airways narrowing. The presence of both these signs together in addition to wheezes argues for underlying obstructive airway disease as the etiology of SOB rather than, for example, heart failure or pulmonary embolism. Pulmonary function test is very useful in evaluation of patients with wheezes but usually it is not readily available at the bedside and needs good patient cooperation to get accurate results. These two signs exemplify the value of a simple yet valuable part of physical examination, observation, a part that physicians tend to ignore sometimes with the trend and habit to listen to heart and lung sounds over patient’s clothes.

## “HINTS” examination for vertigo

A neuro-ophthalmologist described in an interesting paper ( Kattah, 2009) a three-step oculomotor tests that challenged MRI in picking up early posterior circulation stroke. These maneuvers (head impulse, direction-changing nystagmus, and alternate eye cover tests) can be done in a couple of minutes to evaluate patients presenting with one of the common symptoms that internists encounter: dizziness and vertigo. The authors of this study showed that these 3 tests, when done appropriately, have a very high sensitivity to pick up acute infarction as a cause of acute vestibular symptoms (e.g. vertigo, dizziness, nausea and vomiting, etc.) in patients who present with these symptoms and have at least one risk factor for stroke. MRI done within 48 hours of presentation did miss 8 cases of these strokes and those were shown in repeat MRI to be posterior circulation strokes (5 lateral medullary infarcts, 1 lateral pontomedullary infarct, and 3 cerebellar infarcts).

Hospitalists, together with emergency physicians, are usually the first to evaluate patients with acute dizziness and vertigo in the hospital. Making the diagnosis of stroke out of the other differential diagnoses can be challenging in this setting since dizziness is a very nonspecific symptom with wide differential diagnosis. In the right clinical circumstances and with accurate focused bedside exam, more expedited work-up can be initiated for consideration of tissue plasminogen activator (tPA) administration if acute ischemia was discovered early enough.

## Mental status exam for delirium

Acute confusional state or delirium is very common on the general medical wards and even more common in patients admitted to the intensive care unit. Despite that, it is still under recognized and under treated by physicians, especially the hypoactive subtype of delirium. No wonder it should be considered as medical emergency because of its association with higher morbidity, mortality, cost, and length of stay, and hence it is a strong predictor of poor patient outcome. Nevertheless, there is no single diagnostic test for it and diagnosis is usually made by careful bedside evaluation.

Delirious patients can have a variety of presentations, like disorientation, hallucination, and delusions, but decreased attention span and concentration is considered the higher brain function that’s mostly jeopardized and this is important for diagnosis. A couple of simple bedside verbal tasks if performed correctly by patients suspected to have acute confusional state will make the diagnosis very unlikely. Asking the patient to repeat “LUNCH” backwards, or name six months backwards were included in an emergency department (ED) protocol to screen old patients for delirium. This simple protocol, called the delirium triage screen (DTS), was found to have a likelihood ratio negative (LR-) of 0.04 and so was very useful to rule out delirium in old patients presenting to ED ( Han, 2013).

## Physical exam and modern technology are partners, not rivals

Good history taking skills and the art of careful physical examination, I believe, will remain the foundation for making good clinical sense out of patients’ symptoms. Delving into literature, one can find that many physical examination maneuvers and signs can still be called survivors in this era of modern technology and diagnostic evolution. We are blessed to witness this blossoming renaissance of diagnostic technology evolving into our clinical practice. But if this technology is used inappropriately, this will add to the already burdensome cost of medical care that we face currently, without the comparative excellent clinical outcome that we all strive for. Most of these tests also are not without their risks which need to be discussed with patients.

By knowing the clinical relevance of history questions and the reliability of physical findings and their likelihood ratios for specific diagnosis, internists can have a strong basement for building the most valuable and cost-effective work up for their patients, without wasting resources. Physical exam findings tend to be more useful when combined rather than when considered in isolation. So, even findings that have modest likelihood ratios (e.g. <5) when elicited together in the same patient will strengthen each other and further increase the post-test probability. Add to this the other advantages of careful bedside examination like increasing the rapport and trust between physicians and their patients (without additional cost!) and the benign nature of most physical exam maneuvers at the bedside when performed appropriately. Nevertheless, some examination signs remain to be questioned in terms of their diagnostic power, and need more scrutiny to determine their accuracy in clinical practice.

## Take Home Messages

• Learn the likelihood ratios for the various physical examination maneuvers that you need for your practice;
• Ordering modern diagnostic tests should not replace careful physical examination skills to reach a diagnosis;
• Some important diagnoses can only be made by a careful bedside physical examination.

## Notes On Contributors

Mohammed Gafar Elhassan, MD, FACP, is an Assistant Professor of Medicine and Academic Hospitalist in the Department of Medicine at University of California, San Francisco (UCSF)/Fresno Center for Medical Education and Research. His main clinical interests are physical examination skills and point-of-care ultrasound training.
