# Supplementary material for: Can Physical Examination Skills Survive the Era of Modern Diagnostic Tests?
Source: MedEdPublish (2016). 2017 Jan 9;6:3. [Version 1] doi: 10.15694/mep.2017.000003 (PMC10885214; doi:10.15694/mep.2017.000003)
Supplement: Peer review history

# Reviewer report: Reviewer response for version 1

**DOI:** 10.21956/mep.19414.r28499

**Published:** 2017-02-17

**Authors:** P Ravi Shankar

**Affiliations:** 1 American International Medical University

This review has been migrated. The reviewer awarded 4 stars out of 5 This is an interesting manuscript examining the relevance of selected physical examination skills in the present era. In today’s medicine dominated by technology and with the emphasis on maximizing profits physical examination is becoming much less common. When I went to medical school in South India there was a strong emphasis on physical examination. I agree with the author that physical examination can in certain instances preclude the ordering of expensive investigations and can reduce cost. The author has used evidence from studies in the literature examining the likelihood ratio and the evidence for selected physical examination skills. This is a mostly well-written manuscript but corrections can be carried out in a few places.

Reviewer Expertise:

NA

No decision status is available

# Reviewer report: Reviewer response for version 1

**DOI:** 10.21956/mep.19414.r28501

**Published:** 2017-02-05

**Authors:** Trudie Roberts

**Affiliations:** 1 Leeds Institute of Medical Education

This review has been migrated. The reviewer awarded 3 stars out of 5 An interesting contribution but I'm not sure I can agree with the third heart sound section. In future the use of point of contact ultrasound will mean that directly visualising the heart and its function will become increasingly important. I know people will disagree but a considerable number of reputable cardiologists rarely use a stethoscope these days. Students can be taught to obtain excellent four chamber views of the heart in around 90 mins of introducing the technology. Interpretation for common conditions can be taught in a similar time. Now hand held ultrasound devices are increasingly available and the next generation will be a probe attached to a smart phone then this technology will become increasingly available globally. There will be resistance to this technology of course as there was to Laennec's invention 200 years ago ....

Reviewer Expertise:

NA

No decision status is available

# Reviewer report: Reviewer response for version 1

**DOI:** 10.21956/mep.19414.r28498

**Published:** 2017-01-27

**Authors:** Thomas Puthiaparampil

**Affiliations:** 1 UNIVERSITI MALAYSIA SARAWAK

This review has been migrated. The reviewer awarded 3 stars out of 5 History taking and physical examination are the cornerstones of traditional medical practice. I congratulate the author for highlighting this unglamorous aspect of medicine in this modern era, when such basic aspects of patient care are given less importance or rather neglected. As the author has mentioned, physical examination skills have declined in recent years with the onslaught of investigations. It is rightly pointed out that many of the expensive and harmful investigations could be avoided, if proper history taking and physical examinations are practiced. I find crico-sternal distance and tracheal tug more useful as signs of chronic airway diseases with emphysema. Another useful observation in such conditions is the inspiratory recession of soft tissues in the supraclavicular fossae and the hypertrophic and overactive accessory muscles of respiration. Above all, spending some quality time for history taking and physical examination would help to create a rapport with the patient, which would surely avoid unnecessary litigations.

Reviewer Expertise:

NA

No decision status is available

# Reviewer report: Reviewer response for version 1

**DOI:** 10.21956/mep.19414.r28503

**Published:** 2017-01-20

**Authors:** sathyanarayanan varadarajan

**Affiliations:** 1 SRM Medical College, SRM University

This review has been migrated. The reviewer awarded 4 stars out of 5 The author of this article believes that Good history taking skills and the art of careful physical examination will remain the foundation for making good clinical sense out of patients’ symptoms. In support of this, the author skillfully highlighted some of the easy to perform physical examination signs, the third heart sound; maximal tracheal height and Hoover’s sign; the “HINTS” examination for patients with vertigo and simple bedside questions to screen for delirium. Through these examples the author conveys the message that these kinds of simple skills might help what to focus on during the patient encounter and also to have a strong basement for building the most valuable and cost-effective work up for their patients, without wasting resources. He also feels they might increase the rapport and trust between physicians and their patients. The author strongly concludes that ordering modern diagnostic tests should not replace careful physical examination skills to reach a diagnosis and some important diagnoses can only be made by a careful bedside physical examination.This article is a must read for all the clinicians and medical educators who feel the importance of learning and teaching physical examination skills in the era of modern diagnostic tests.

Reviewer Expertise:

NA

No decision status is available

# Reviewer report: Reviewer response for version 1

**DOI:** 10.21956/mep.19414.r28497

**Published:** 2017-01-10

**Authors:** Richard Hays

**Affiliations:** 1 James Cook University

This review has been migrated. The reviewer awarded 3 stars out of 5 I found this paper interesting because it broadens the agenda of clinical skills teaching. In our current 'simulate and practice safely first' approach, what is taught is sometimes influenced heavily by what is available in the various simulation models. Learning the clinical skills suggested here probably relies on 'old fashioned' demonstration by experienced clinicians on real patients, followed by substantial clinical experience, including in urgent care settings, to practice the skills and develop judgement. Nothing wrong with this approach of course, but should simulation development be guided by what is needed or what is possible/easy/relatively inexpensive? If the Skillslab could simulate more before genuine e clinical exposure, perhaps learning would be broader and deeper?

Reviewer Expertise:

NA

No decision status is available

# Reviewer report: Reviewer response for version 1

**DOI:** 10.21956/mep.19414.r28500

**Published:** 2017-01-09

**Authors:** Gary D. Rogers

**Affiliations:** 1 Griffith University

This review has been migrated. The reviewer awarded 3 stars out of 5 This was an interesting reflection on the value of physical examination signs with examples from a diverse range of specialty areas. It would have been useful for the casual reader to include a short explanation of the meaning of Likelihood Ratios, since these are considered extensively in the text and are not commonly understood. It would also have been useful to reference the excellent book on this topic by Simel and Rennie (2009). The other important issue to which the author alludes a little, but which warrants robust discussion among clinicians and educators is which physical examination manoeuvres we should encourage our students to *stop* doing because they are of no diagnostic value or even misleading.

Reviewer Expertise:

NA

No decision status is available

# Reviewer report: Reviewer response for version 1

**DOI:** 10.21956/mep.19414.r28502

**Published:** 2017-01-09

**Authors:** Trevor Gibbs

**Affiliations:** 1 AMEE

This review has been migrated. The reviewer awarded 4 stars out of 5 An interesting personal opinion to read. Although I am unsure if the use of sound physical examinations are getting less, I do agree that there appears to be a general trend in relying upon diagnostic / laboratory / technological tests to make a diagnosis rather than support it- almost "short-cutting" the arrival to a diagnosis. I think that the author has used some common and useful examples to support his opinion and also by using the appropriate statistics, has made this a very useful paper for all clinicians to read and consider

Reviewer Expertise:

NA

No decision status is available
